# Nontraumatic Ulnar Artery Pseudoaneurysm in the Forearm of a Seven-Month-Old Infant: A Case Report

**DOI:** 10.7759/cureus.99334

**Published:** 2025-12-15

**Authors:** Yoshiyuki Hara, Kenjiro Hasegawa, Satoe Arai, Tomoyuki Noda

**Affiliations:** 1 Department of Orthopedic Surgery, Kawasaki Medical School General Medical Center, Okayama, JPN; 2 Department of Plastic and Reconstructive Surgery, Kawasaki Medical School General Medical Center, Okayama, JPN

**Keywords:** aneurysm, infant, nontraumatic, reconstruction, ulnar artery

## Abstract

Ulnar artery aneurysms are uncommon in infants and even in adults. It is difficult to diagnose, with a range of different treatment methods. To the best of our knowledge, no case has been reported that occurred in the forearm of infants to date. We report a rare case of a nontraumatic ulnar artery pseudoaneurysm in the forearm of a seven-month-old infant. It was diagnosed by ultrasound and contrast-enhanced computed tomography and treated by aneurysm resection and reconstruction. The early postoperative course has been uneventful, but long-term follow-up will be required as the patient grows.

## Introduction

Aneurysms occur when the laminar structure of the vascular wall is weakened, causing it to bulge. They may be true aneurysms or pseudoaneurysms. Pseudoaneurysms are often complications of penetrating trauma, whereas true aneurysms often occur following blunt trauma [1]. 

Aneurysms in the upper arm are uncommon, and they are almost always pseudoaneurysms [2,3]. Most reported ulnar artery aneurysms in adults are cases of hypothenar hammer syndrome in the palm [4-8], and a few cases of true aneurysms in the palms of children have also been reported [5]. We report a rare case of a pseudoaneurysm of the ulnar artery in the distal forearm of an infant with no history of trauma.

## Case presentation

**Figure 1:**
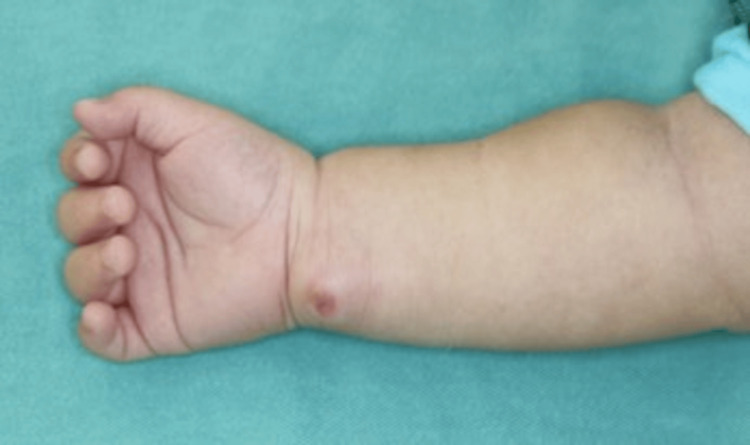
External examination on presentation in our department. A mass is evident on the ulnar side of the right distal forearm.

A seven-month-old male infant with no developmental issues was brought to our hospital’s Department of Pediatrics after his mother noticed a swelling in his right distal forearm with no known cause. His parents were unaware of any traumatic injury, such as a blow or puncture. There was a palpable pulsating mass on the ulnar side of the right distal forearm (Figure 1), and ultrasound showed a mass that was continuous with the ulnar artery.

Compression treatment was tried, but since there was no reduction in the size of the mass after seven days, the infant was referred to our department. Contrast-enhanced computed tomography (CT) showed an ulnar artery aneurysm measuring 21×13×11 mm, but no radial artery hypoplasia or other vascular anomaly was evident.

**Figure 2:**
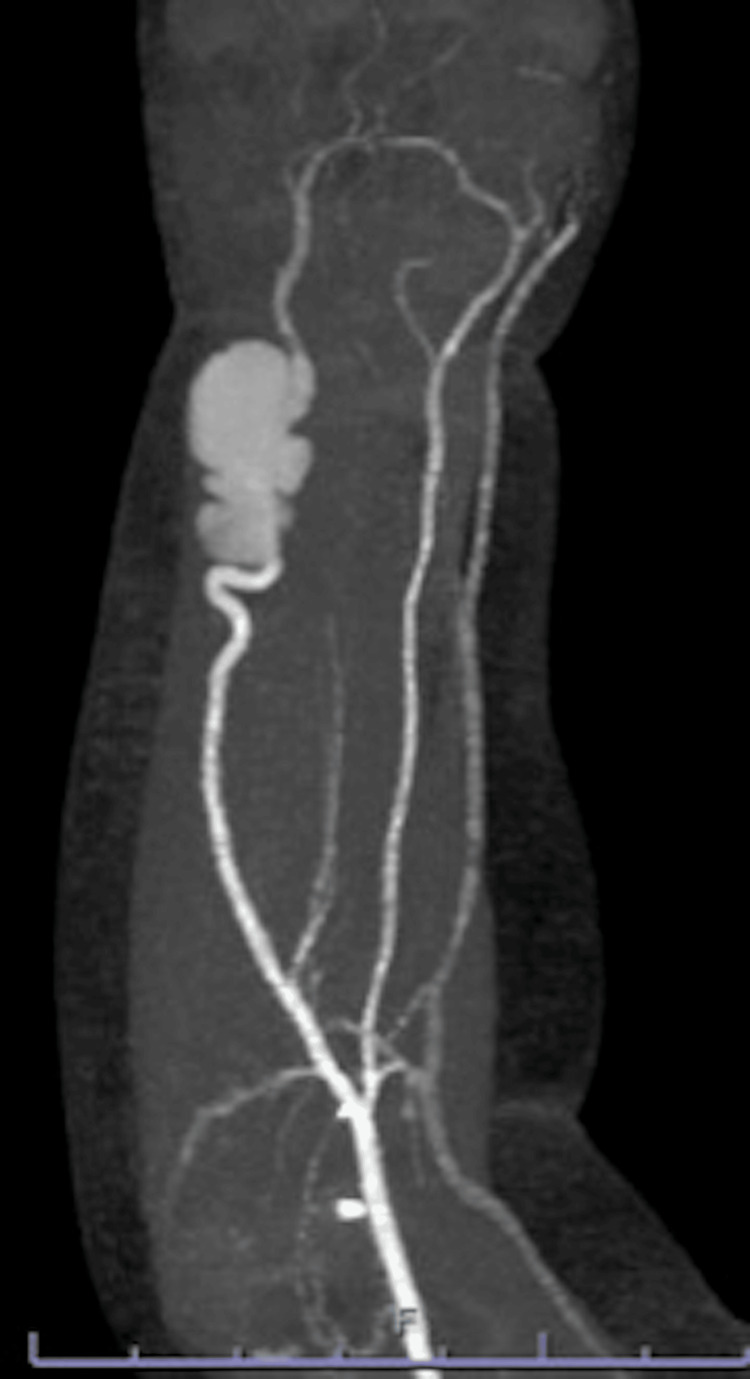
Preoperative contrast-enhanced CT of the right forearm. An ulnar artery aneurysm is evident. There is no sign of radial artery hypoplasia or other vascular anomaly.

**Figure 3:**
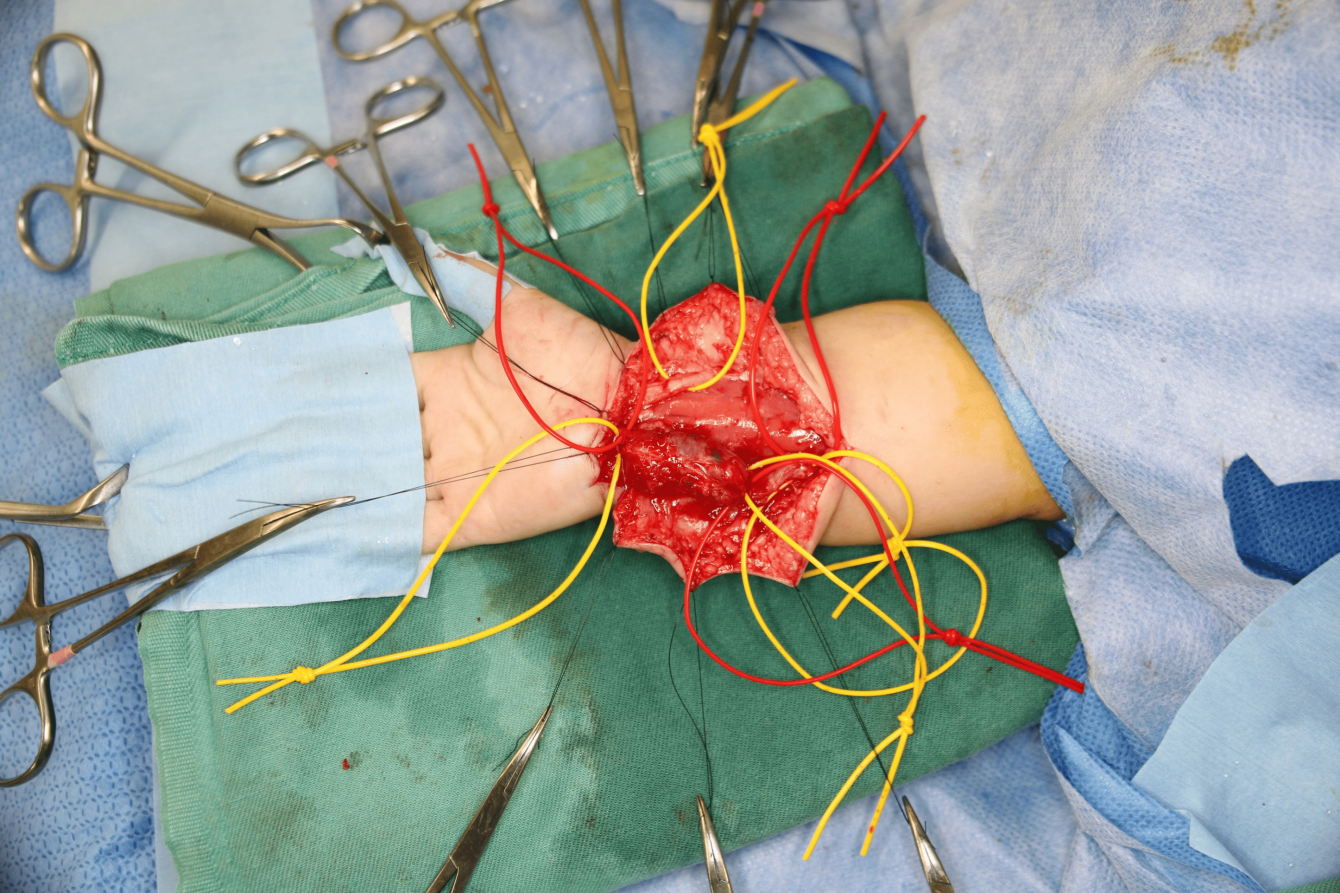
Intraoperative view of the right forearm The median and ulnar nerves are identified, and the ulnar artery aneurysm is confirmed.

Surgery was performed in our department the following day to avoid further impact on the aneurysm that could lead to rupture by using hands. During the operation, the aneurysm was first confirmed (Figure 3) and then resected.

**Figure 4:**
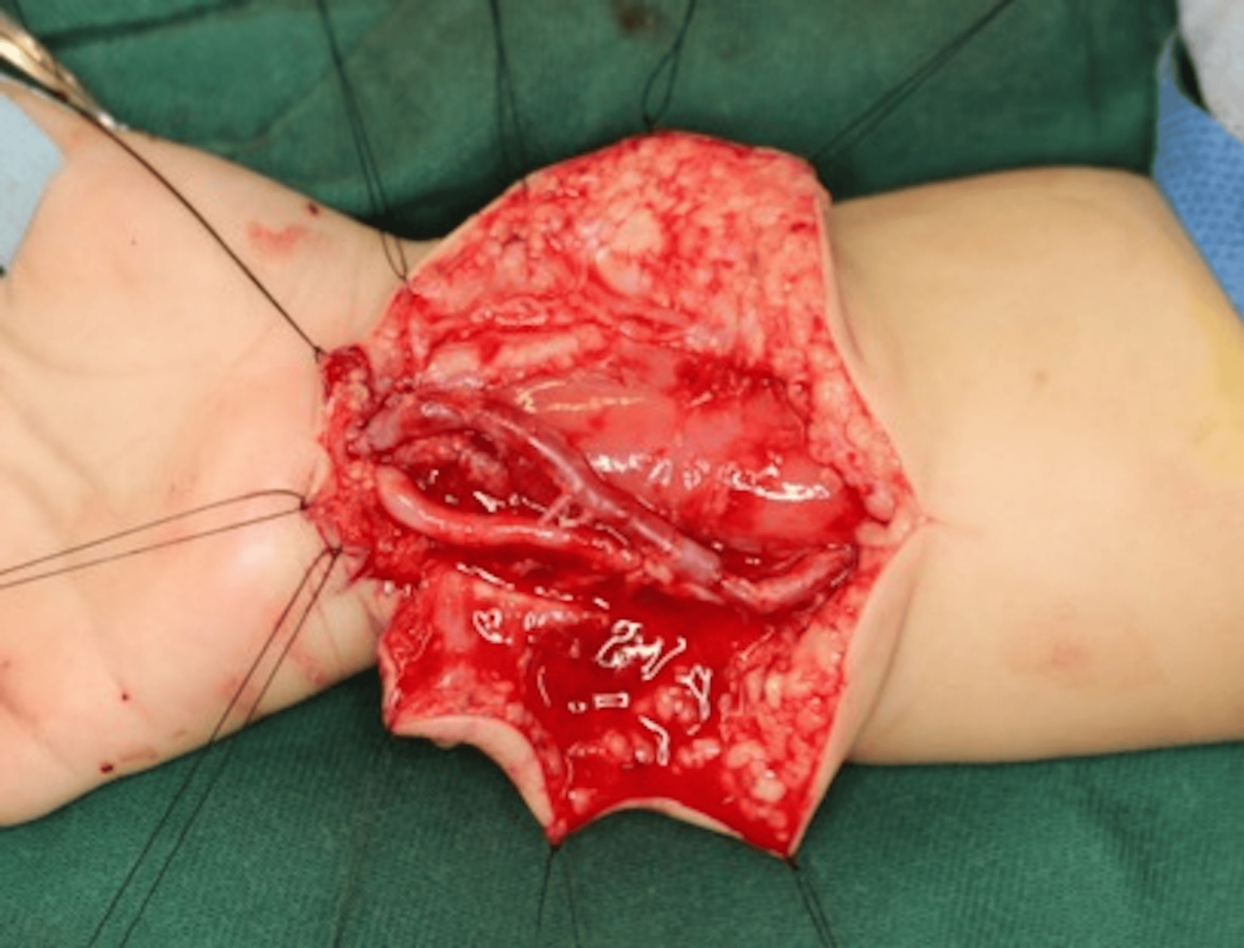
Intraoperative view after ulnar artery reconstruction. The ulnar artery is revascularized using a greater saphenous vein graft.

After this resection, there was no discoloration of the hand or other signs suggestive of impaired perfusion. A graft was then harvested from the greater saphenous vein in the right thigh and used to reconstruct the ulnar artery (Figure 4).

**Figure 5:**
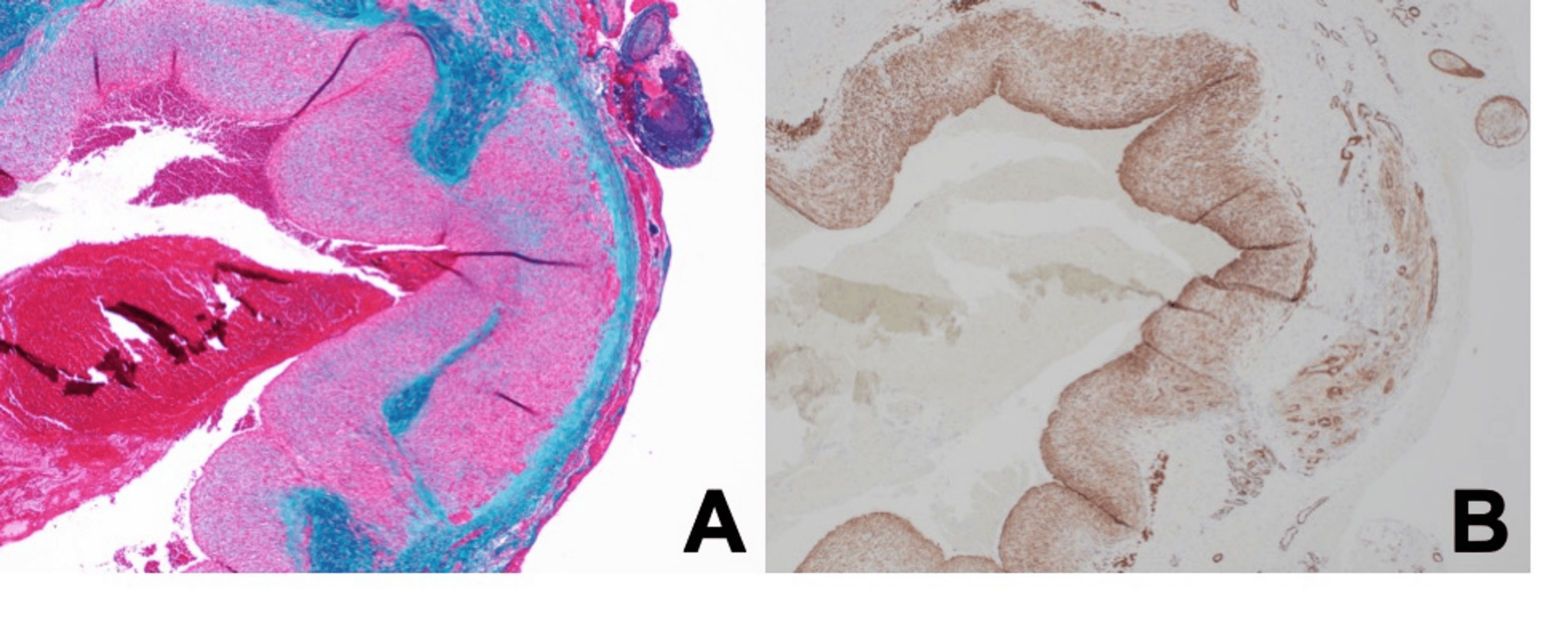
Histopathological investigation of the aneurysm (4× objective) There is no sign of infiltration by eosinophils or inflammatory cells. In the vascular wall, thickening of the intima, thinning of the internal tunica elastica, and obscuration of the smooth muscle of the tunica media are apparent, which are signs of a pseudoaneurysm. (A) E-Masson staining. (B) α-SMA immunostaining.

Histopathological investigations of the resected aneurysm did not show infiltration by eosinophils or inflammatory cells, and thickening of the intima, thinning of the internal lamina elastica, and obscuration of the smooth muscle of the tunica media were observed, which are signs of a pseudoaneurysm (Figure 5).

**Figure 6:**
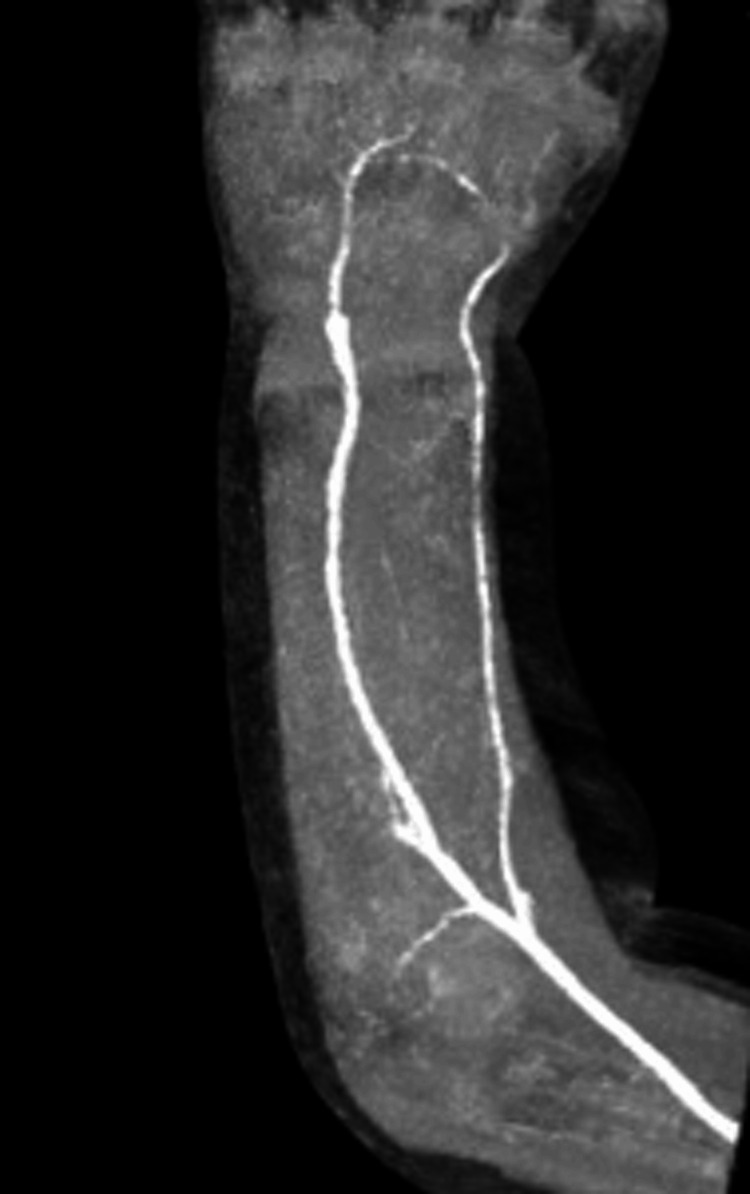
Contrast-enhanced CT of the right forearm four months after surgery. The reconstructed ulnar artery is patent, and there is no impairment of blood flow to the hand.

Four months postoperatively, the reconstructed ulnar artery was patent (Figure 6), and there was no impairment of blood flow to the hand, wrist, and finger movements were also normal.

## Discussion

Aneurysms occurring in peripheral arteries in the upper limbs are rare, and their presence in the forearm and palm is reported to account for only around 10-15% of such cases [2]. Aneurysms are divided into true aneurysms and pseudoaneurysms, with pseudoaneurysms caused by sharp trauma, more commonly occurring in the limbs [3].

The incidence of ulnar artery aneurysms has yet to be established, but in adults, the most frequently reported form is a true aneurysm associated with hypothenar hammer syndrome caused by blunt trauma to the palm [4]. Nontraumatic causes include radial artery hypoplasia and other vascular anatomical anomalies, Behçet’s disease, rheumatoid vasculitis, and connective tissue diseases such as Marfan’s syndrome and Kawasaki disease, whereas a few cases associated with hepatitis B and C have also been reported [6].

As far as we are aware, only 14 cases of ulnar artery aneurysm in childhood have been reported previously [1,5,7-18], with the youngest of these being a five-month-old infant [7]. In all previous cases, the aneurysm was a true aneurysm, was located in the palm, and there has been no reported case of one occurring proximal to the wrist [1,5,7-18]. Two were congenital [5,13], one was of unknown cause [9], two were non-traumatic [10,18], and all the others were caused by trauma. No case was similar to the present patient, in which a pseudoaneurysm believed to be of nontraumatic origin occurred in the ulnar artery in the distal forearm of an infant, has been reported previously.

Aneurysms are diagnosed based on the location of a pulsatile mass. Unlike in the present patient, however, they are often unclear, and caution is therefore required. Doppler ultrasound is a useful noninvasive examination, but it cannot always provide a definitive diagnosis [7]. The present patient was a seven-month-old infant who was unable to hold still during the examination, which yielded only the size of the mass and the fact that it was continuous with the ulnar artery. In adults, angiography is helpful for the evaluation of ulnar artery aneurysms. However, this requires arterial puncture, and small children must also be sedated during the procedure. Magnetic resonance angiography (MRA) is also a useful diagnostic tool that is capable of distinguishing between aneurysms and similar-looking swellings or soft tissue tumors and can be used for comparisons with the surrounding soft tissue [10], but since small children must also be sedated during this procedure, it usually takes longer than contrast-enhanced CT. In the present patient, the mass was located in the ulnar artery in the distal forearm, and since preoperative ultrasound had shown that it was continuous with the ulnar artery, it was considered necessary to compare the blood flow in the radial and ulnar arteries. Despite the requirements for sedation and radiation exposure, contrast-enhanced three-dimensional CT (3DCT) was therefore performed. This was helpful for diagnosis, providing both the location and size of the mass and enabling a comparison with the radial artery.

There are no clinical guidelines for ulnar artery aneurysms in children, and whether to treat them solely by resection or to conduct repair or reconstruction as well is a matter of debate. Moosa et al. recommend performing repair or reconstruction whenever possible [8], whereas Iyer et al. state that they do not carry out repair or reconstruction in the absence of symptoms of ischemia after the aneurysm has been removed [7]. However, both reports concerned aneurysms in the palm, and when they are located proximal to the wrist, as in the present patient, if the artery is not reconstructed after resection, then perfusion to the hand will only be provided by the radial artery, meaning that it will necessarily decrease. Although perfusion to the hand via the radial artery alone is considered sufficient, repair or revascularization with a venous or arterial graft in addition to aneurysm resection may be necessary in children to minimize the future risk of ischemia [9].

In the present case, resecting the aneurysm left a large defect, so that even though there were no signs suggestive of ischemia in the hand at that point, revascularization was performed with a venous graft for the reason outlined above. Four months after the surgery, the patient’s course had been uneventful with no recurrence of the aneurysm, but further long-term follow-up will be required.

## Conclusions

This was a rare presentation of an ulnar artery pseudoaneurysm that we encountered in the forearm of an infant. It was a nontraumatic, and we opted for its excision and reconstructed the ulnar artery. The decision of reconstruction was made because of the very young age of this patient and to minimize the risk of ischemia in the future.

## References

[1] Odajima R, Nishimoto S, Kawai K, Ishise H, Kakibuchi M (2021). A traumatic ulnar artery aneurysm in an infant. J Surg Case Rep.

[2] Shohei Y, Shinsuke M, Hiroyuki O (2001). Two case reports of the traumatic radial artery aneurysms (Article in Japanese). Geka.

[3] Gray RJ, Stone WM, Fowl RJ, Cherry KJ, Bower TC (1998). Management of true aneurysms distal to the axillary artery. J Vasc Surg.

[4] Ferris BL, Taylor O, kayama K (2000). Hypothenar hammer syndrome: proposed etiology. J Vasc Surg.

[5] Kishi Y (2020). A case of congenital aneurysm of the ulnar artery of the palm. Plast Aesthet Res.

[6] Mazzaccaro D, Malacrida G, Stegher S, Occhiuto MT, Caldana M, Tealdi DG, Nano G (2012). Ulnar artery aneurysm: case report and review of the literature. G Chir.

[7] Iyer RS, Hanel DP, Enriquez BK, Weinberger E (2012). Ulnar artery aneurysm causing palmar mass in 5-month-old girl. Pediatr Radiol.

[8] Moosa MA, Shaikh SA, Sophie Z (2019). Rare presentation of an ulnar artery aneurysm in a six-month-old baby: case discussion. Ann Vasc Dis.

[9] Meals CG, Carey GB, Higgins JP, Chang B (2017). Ulnar artery aneurysm in 6-month-old: a case report. Hand (N Y).

[10] Deune EG, McCarthy EF (2005). Reconstruction of a true ulnar artery aneurysm in a 4-year-old patient with radial artery agenesis. Orthopedics.

[11] Rikukawa H, Kudo T, Takahashi K, Muramatsu T, Nakazato T, Sakata K, Sezai Y (1992). A case report of true palmar aneurysm (Article in Japanese). Nihon Geka Gakkai Zasshi.

[12] Martin RD, Manktelow RT (1982). Management of ulnar artery aneurysm in the hand: a case report. Can J Surg.

[13] Offer GJ, Sully L (1999). Congenital aneurysm of the ulnar artery in the palm. J Hand Surg Br.

[14] Witt PD, Bowen KA, Johansen K (2003). True ulnar artery aneurysm of the hand in an 8-year-old boy. Plast Reconstr Surg.

[15] Al-Omran M (2007). True ulnar artery aneurysm of the hand in an 18-month-old boy: a case report. J Vasc Surg.

[16] Parsa AA, Higashigawa K, Parsa FD (2008). Arterial aneurysms of the hand. Hawaii Med J.

[17] Amjad I, Murphy T, Zahn E (2010). Diagnosis and excision of an ulnar artery aneurysm in a two-year-old boy. Can J Plast Surg.

[18] Stalder MW, Sanders C, Lago M, Hilaire HS (2016). Multilocular true ulnar artery aneurysm in a pediatric patient. Plast Reconstr Surg Glob Open.

